# An iron-based green approach to 1-h production of single-layer graphene oxide

**DOI:** 10.1038/ncomms6716

**Published:** 2015-01-21

**Authors:** Li Peng, Zhen Xu, Zheng Liu, Yangyang Wei, Haiyan Sun, Zheng Li, Xiaoli Zhao, Chao Gao

**Affiliations:** 1 MOE Key Laboratory of Macromolecular Synthesis and Functionalization, Department of Polymer Science and Engineering, Zhejiang University, Polymer Building, 38 Zheda Road, Hangzhou 310027, P.R. China

## Abstract

As a reliable and scalable precursor of graphene, graphene oxide (GO) is of great importance. However, the environmentally hazardous heavy metals and poisonous gases, explosion risk and long reaction times involved in the current synthesis methods of GO increase the production costs and hinder its real applications. Here we report an iron-based green strategy for the production of single-layer GO in 1 h. Using the strong oxidant K_2_FeO_4_, our approach not only avoids the introduction of polluting heavy metals and toxic gases in preparation and products but also enables the recycling of sulphuric acid, eliminating pollution. Our dried GO powder is highly soluble in water, in which it forms liquid crystals capable of being processed into macroscopic graphene fibres, films and aerogels. This green, safe, highly efficient and ultralow-cost approach paves the way to large-scale commercial applications of graphene.

## Abstract (web summary)

Oxidants currently used in the preparation of graphene oxide (GO) require long reaction times, relatively high temperatures and costly waste treatment. Here, the authors propose the use of a strong green oxidant, K2FeO4, establishing a fast, safe, toxicity-free route to GO production.

## Graphical abstract

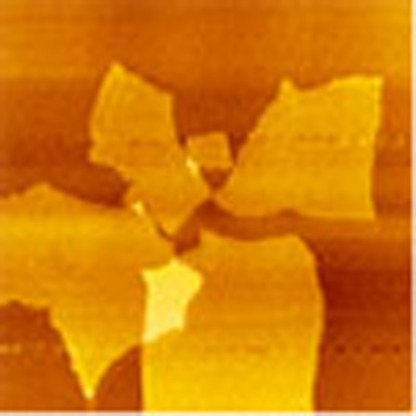

Graphene has been the focus of significant attention for its potential across a broad spectrum of applications due to its unrivalled mechanical, electrical and thermal properties^1^,^2^,^3^. Thus far, two main strategies have been developed for the production of graphene from graphite: mechanical exfoliation (including solvent and ultrasonic-assisted methods)^4^,^5^,^6^ and chemical oxidation–reduction^7^,^8^,^9^,^10^,^11^,^12^,^13^,^14^,^15^,^16^,^17^,^18^. Mechanically exfoliated graphene possesses few or no defects^6^,^19^, but suffers from poor solubility (<0.1 mg ml^−1^)^4^ and extremely low productivity (for example, ~2.0 × 10^−3^ g h^−1^)^20^. In addition, because of strong π–π stacking, such graphene is prone to irreversible aggregation after concentration and drying.

A recently applied process of high rate-shear exfoliation in N-methyl-2-pyrrolidone provides notable increases in productivity (~5.3 g h^−1^ (ref. 19), still far too low for commercial needs); however, the addition of polymer surfactants is necessary, otherwise the pristine graphene would aggregate and precipitate. Such graphene sheets are a mixture of different layers, limiting experimental reproducibility and inhibiting its use in fine applications. By comparison, preparation by chemical oxidation yields highly soluble single-layer graphene oxide (slGO; solubility>110 mg ml^−1^)^21^ in large-scales (up to tons scale), enabling easy processing of slGO into high performance composites and macroscopic materials such as fibres^22^,^23^, films/papers^24^ and aerogels^25^ by solution-based polymer-type techniques. Although slGO is often denounced for containing defects^16^ that may influence its properties, such defects can be easily repaired through simple chemical reduction^26^. Thermal treatment has the capacity to restore the chemically converted graphene (CCG) back to a material with ultrahigh electrical conductivity (1.83 × 10^5^ S m^−1^) and thermal conductivity (1434 W m^−1^ K^−1^)^27^. These values are far higher than those of mechanically exfoliated defect-free graphene (2.2 × 10^4^ S m^−1^, 313 W m^−1^ K^−1^)^28^. For these reasons, the slGO–CCG route is the more attractive of the two for the industrial production of graphene.

Generally, GO is prepared by the ultrasonic exfoliation of graphite oxide^29^. The preparation methods of GO can be classified by the oxidant employed as either the KClO_3_-based Brodie–Staudenmaier^8^,^9^,^10^ method or the KMnO_4_-based Hummers method^11^,^12^,^13^,^14^. The KClO_3_-based method was first introduced by Brodie^8^ in 1859, modified by Staudenmaier^9^ in 1898 and again modified by Hofmann^10^ in 1937. The reaction medium for this process is nitric acid, which presents the inherent disadvantages of explosion risk, release of hazardous gases (for example, NO_*X*_ and ClO_2_) and the generation of carcinogenic ClO^−^. The Hummers method was first reported in 1958 (ref. 11). Although the change of oxidant circumvented a number of KClO_3_-based issues, it is plagued by the necessity of polluting heavy metal ions (Mn^2+^) and the explosion risk that accompanies the unstable Mn_2_O_7_ intermediates^30^. Various modifications involving minor optimization of the Hummers method have been employed for the synthesis of GO; however, no significant improvements have been made despite the intensive interest in this material^1^,^7^,^31^. In addition, the two methodologies used to obtain slGO require long reaction times (6 h–5 days), relatively high temperatures (>50 °C) and often additional intercalation and ultrasonication processes. These shortcomings result in a costly process in terms of time and energy, a complicated fabrication procedure and carry high costs related to waste treatment. Hence, a green (free of toxic gases and polluting heavy metals), safe (no explosive risk), ultrafast and low cost methodology is eagerly sought.

Herein, we propose a strong yet green oxidant, K_2_FeO_4_, and establish an ultrafast, safe and non-toxic methodology for the scalable production of slGO. The entire fabrication process requires only 1 h, and the as-prepared large GO sheets are nearly 100% single layered without any ultrasonic treatment. Our slGO has a similar chemical structure and solubility to materials prepared using the conventional long-time modified Hummers method. Furthermore, the GO powder obtained by drying slGO solutions can be re-dissolved in water or organic solvents to form stable liquid crystals (LC) and subsequently assembled into macroscopic materials such as one-dimensional (1D) fibres, 2D films and 3D aerogels. In addition, sulphuric acid is recycled in our protocol. Through the refreshing of oxidant, our approach dramatically reduces the effluent and lowers the operating cost. This method paves the way for cheap, eco-friendly, large-scale production of slGO and its macroscopic materials.

## Results

### Selection of oxidant

Oxidant is the most important controlling factor in the preparation of GO. The Brodie–Staudenmaier^8^,^9^,^10^ method and Hummers^11^,^12^,^13^,^14^ method essentially differ in their choice of oxidant. The prevailing oxidants, predominantly KClO_3_ and KMnO_4_, provide high oxygen content to the resultant GO materials; however, their byproducts are highly polluting and intermediates in the processes carry a high risk of explosion. For example, KClO_3_ is a key ingredient in blasting caps and is prone to explode when mixed with combustible materials. It is also frequently used in explosives and fireworks, and is thus strictly controlled in China. In the synthesis of GO with KClO_3_, the toxic and explosive gas ClO_2_ is generated in the concentrated sulphuric acid solvent. In addition, KMnO_4_ is easily converted into Mn_2_O_7_, which is prone to explode above 55 °C in an acidic environment^30^. The use of KMnO_4_ generates massive amounts of the heavy metal pollutant Mn^2+^, which can cause great damage to human and plant life in an ecosystem. The various modifications of these two methodologies over the past decade have not been able to remedy the substantial inherent environmental and safety issues related to the production of toxic gases and heavy metal pollutants or the risk of explosion.

To resolve the problems posed by the conventional methods, an alternative oxidant for GO production is sought. The new oxidant must satisfy the following prerequisites: (1) high oxidation efficiency, (2) no risk of explosion and (3) no toxic or polluting byproducts. After numerous experiments, we identified K_2_FeO_4_ as the novel oxidant of choice. K_2_FeO_4_ is an eco-friendly and highly efficient oxidant with harmless byproducts. Currently, it is widely used in the fields of environmental protection and water treatment^32^,^33^. K_2_FeO_4_ has an electrode potential of 2.2 V, which is much higher than that of KMnO_4_ (1.36 V) in acid environments, and should thereby considerably decrease the required reaction time. As opposed to KMnO_4_, K_2_FeO_4_ can be safely used at temperatures as high as 100 °C, due to the absence of explosive intermediates. In addition, as a commonly used water treatment agent, K_2_FeO_4_ is inexpensive and commercially available. Therefore, K_2_FeO_4_ is attractive as a new-generation oxidant for the preparation of GO in the desired eco-friendly and highly efficient manner.

### Preparation and characterization of GO

**Figure 1:**
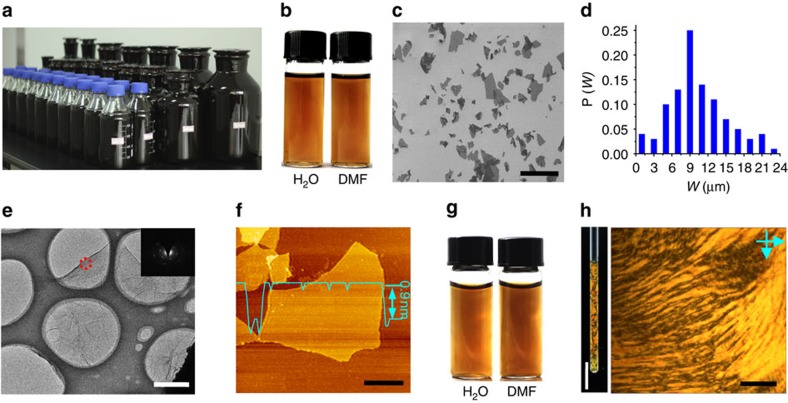
**Large-scale synthesis of single-layer GO**^**Fe**^ **via K**_**2**_**FeO**_**4**_**-based methodology.** (**a**) Seventy-five litre GO^Fe^ aqueous solution with a concentration of 10 mg ml^−1^. (**b**) GO^Fe^ solution in H_2_O and N,N-dimethylformamide (DMF) with a concentration of 3 mg ml^−1^. (**c**) SEM image of GO^Fe^ on Si/SiO_2_ substrate. (**d**) The size distribution of the GO^Fe^ sheets, counted and calculated from **c** and Supplementary Fig. 2. (**e**) TEM image of GO^Fe^ and its SAED diffraction patterns (inset). (**f**) Tapping mode AFM image and height profile of GO^Fe^. (**g**) GO^Fe^ solution of H_2_O and DMF with a concentration of 3 mg ml^−1^ after storage for 1 year. (**h**) Image of aqueous LCs in a quartz tube between crossed polarisers and POM image between crossed polarisers in planar cells of aqueous GO^Fe^ LCs at a concentration of 3 mg ml^−1^. Scale bars, 20 μm (**c**), 2 μm (**e**), 4 μm (**f**) and 5 mm (**h**, left), 1 μm (**h**, right).

Typically, concentrated sulphuric acid, K_2_FeO_4_ and flake graphite were loaded into a reactor and stirred for 1 h at room temperature. The dark green suspension gradually became a grey viscous fluid. After recycling the H_2_SO_4_ reaction medium by centrifugation, the precipitate was purified by repeated centrifugation and water-washing to obtain highly water soluble slGO (solubility>27 mg ml^−1^), coined as GO^Fe^. Because the reaction process is extremely simple and requires no energy transfer (either heating or cooling), it is straightforward to scale up. For instance, we successfully used a 20-l reactor to prepare 750 g of GO^Fe^ in one pot (Supplementary Fig. 1), corresponding to a 75 l GO^Fe^ aqueous solution with a concentration of 10 mg ml^−1^ (Fig. 1a).

The composition of GO^Fe^ was analyzed via combustion analysis, quantitative X-ray photoelectron spectroscopy (XPS) and inductively coupled plasma mass spectrometry (ICP-MS). Combustion analysis showed that GO^Fe^ has a relative composition of CO_0.51_H_0.22_S_0.028_. The XPS spectrum confirmed the composition of GO^Fe^ as follows (at.%): C (68.51%), O (31.14%), S (0.30%), Si (0.03%), N (0.01%), P (0.01%). ICP-MS measurements demonstrated the existence of negligible metal ion content: Fe (0.13 p.p.m.), Mn (0.025 p.p.m.), Co (0.073 p.p.m.), Cu (0.017 p.p.m.), Pb (0.033 p.p.m.) and Ni (0.014 p.p.m.). Notably, despite the high concentration of K_2_FeO_4_ in the reaction, the negligible iron content in the final GO^Fe^ after purification by the centrifugation/water-washing protocol indicates that no insoluble byproducts, such as Fe_2_O_3_, are generated in the fabrication and post-treatment processes.

The single-layered nature of the GO^Fe^ dispersion was demonstrated via scanning electron microscopy (SEM), transmission electron microscopy (TEM) and atomic force microscopy (AFM; Fig. 1). Under SEM inspection (Fig. 1c), the GO^Fe^ sheets show typical wrinkles, implying fine flexibility in the slGO sheets. According to the statistics from the SEM images, the GO^Fe^ sheets have a number-average width of ~10 μm and 53% of the relative size distribution (*σ*_w_; Fig. 1d). TEM image also shows an abundance of wrinkles (Fig. 1e), and the selected area electron diffraction patterns (the insert) indicate its single-layer character^34^. The thickness of the GO^Fe^, as measured by AFM, is ~0.9 nm (Fig. 1f), which confirms the single-layered state and the presence of oxygen-containing functional groups on the basal plane^17^.

**Figure 2:**
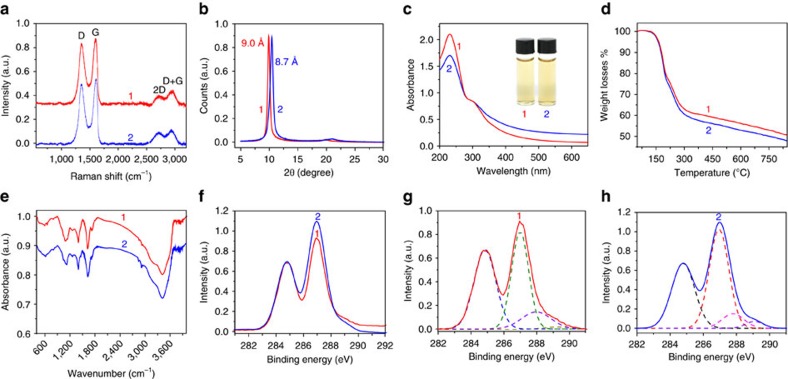
**Comparison of GO**^**Fe**^ **and GO**^**Mn**^. (**a**) Raman spectra recorded using 514 nm laser excitation, (**b**) XRD spectra, (**c**) ultraviolet–visible spectra recorded in aqueous solution at 0.05 mg ml^−1^, (**d**) TGA plots, (**e**) Fourier transform infrared spectra and (**f**–**h**) XPS spectra and its C1s XPS spectra of GO^Fe^ and GO^Mn^. **1** and **2** denote GO^Fe^ and GO^Mn^, respectively. All of these data show that GO^Fe^ and GO^Mn^ have similar composition and structures.

Raman spectra, X-ray diffraction and ultraviolet–visible spectra show that the GO^Fe^ has a similar structure to GO prepared by the modified Hummers method^24^ using KMnO_4_ as the oxidant (GO^Mn^; average lateral size=8 μm; *σ*_w_=79%, Supplementary Fig. 3). The Raman spectrum (Fig. 2a) of GO^Fe^ shows the typical D peak (1,353 cm^−1^), G peak (1,600 cm^−1^), 2D peak (2,698 cm^−1^) and D+G peak (2,945 cm^−1^) with an *I*_D_/*I*_G_ intensity ratio of 0.93, confirming lattice distortions induced by oxidation^16^. The XRD curve of the vacuum-assisted filtration paper indicates that the interlayer spacing of GO^Fe^ is ~9.0 Å (Fig. 2b), which is similar to that of GO^Mn^ (8.7 Å). The ultraviolet/Vis spectra of both GO^Fe^ and GO^Mn^ present a strong absorption peak at 230 nm (π→π* transitions of the conjugation domains) and a weak shoulder peak at ~300 nm (n→π* transitions of the carbonyl groups; Fig. 2c), revealing their similar domain structures^35^.

The thermogravimetric analysis (TGA) profiles of both GO^Fe^ and GO^Mn^ show similar weight loss plots (48–50% mass loss at 800 °C, Fig. 2d). The Fourier transform infrared spectra identify the same functional groups in GO^Fe^ as GO^Mn^ (Fig. 2e): O–H stretching vibrations (3,412 cm^−1^), C=O stretching vibration (1,726 cm^−1^), C=C from sp^2^ bonds (1,624 cm^−1^), O–C–O vibrations (1,260 cm^−1^) and C–O vibration (1,087 cm^−1^). As shown in Fig. 2f–h, the XPS spectra confirm the presence of similar chemical bonds in both GO^Fe^ and GO^Mn^: C=C (284.86 eV), epoxy/hydroxyls (C–O, 287.0 eV), C=O (288.0 eV) and O–C=O (289.2 eV) (ref. 21).

The oxygen-rich functional groups impart a high zeta potential to GO^Fe^ (−58 mV) and excellent solubility in both water and polar organic solvents, as is the case for GO^Mn^ (Fig. 1b). The GO^Fe^ solution retains a homogenously dispersive constitution, without any precipitate, even after storage for 1 year at a concentration of 3 mg ml^−1^ in water or N,N-dimethylformamide (Fig. 1g). The excellent solubility of the highly asymmetrical GO sheets may enable the formation of a lyotropic LC^23^,^36^ phase, which is a criterion used to evaluate the ‘true’ solubility of graphene derivatives. Our GO^Fe^ aqueous dispersions display the vivid textures typical of nematic LCs between crossed polarizers (Fig. 1h).

### Recycling and post-treatment of sulphuric acid

In addition to the problems of polluting heavy metals, toxic gases and tedious reaction times associated with the conventional methods, another persistent criticism of GO production is the pollution associated with the use of concentrated sulphuric acid, the disposal of which significantly adds to the costs of GO. We resolved this issue by recycling concentrated sulphuric acid, a process which was enabled by the strong oxidation ability of K_2_FeO_4_. We recycled the concentrated sulphuric acid at least 10 times without change to the fabrication efficiency (1 h) or the GO quality. Notably, even if the collected sulphuric acid was not immediately reused, its removal proved greatly beneficial to the subsequent GO purification by either centrifugation or sieving/filtration, as well as to the subsequent waste treatment steps. The small amount of H_2_SO_4_ complexed to K_2_SO_4_ and Fe_2_(SO_4_)_3_ in the washing water was neutralized with ammonia, forming mixtures consisting of (NH_4_)_2_SO_4_, K_2_SO_4_ and Fe_2_(SO_4_)_3_, which are used as fertilisers in agriculture. This protocol significantly decreases the cost of slGO.

## Discussion

**Figure 3:**
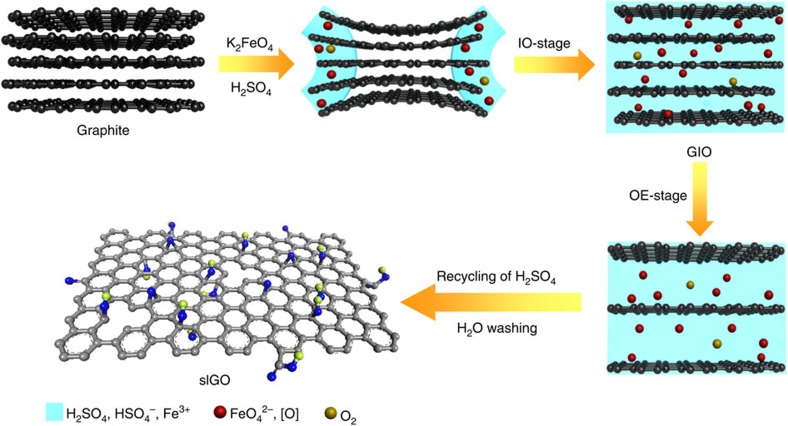
**Mechanism of GO**^**Fe**^ **synthesis with the oxidant of K**_**2**_**FeO**_**4**_. The whole synthetic process (1 h) contains two main stages: intercalation–oxidation (IO) and oxidation–exfoliation (OE). The *in situ* generated FeO_4_^2−^ and atomic oxygen [O] act as oxidants and the O_2_ formed from residual [O] provides mild and durative gas exfoliation. In the IO stage, the concentrated sulphuric acid and oxidants intercalate into the layers of graphite to form intercalated graphite oxide (GIO). During the intercalation, the oxidants break the π–π conjugated structures of graphite, generating negatively charged functional groups, and increasing the interlayer spacing. In the following OE stage, the oxidants further oxidize the carbon basal planes of GIO, giving rise to more functional groups and enlarging the interlayer space. After recycling of sulphuric acid and washing with water, 100% slGO is achieved.

To deeply understand the fast oxidation–exfoliation process in our K_2_FeO_4_-based system, we investigated the effects of oxidation on the dispersive state in water for samples collected at different reaction times. Supplementary Figure 4 shows the dispersion states of the materials after standing for 24 h. Only the solution observed at 1 h of reaction time has no precipitate, implying that the functional group density is high enough to overwhelm the aggregation tendency. Furthermore, the colour of the solutions becomes lighter with increasing oxidation time due to the gradual destruction of π–π conjugate structures by the formation of functional groups. More subtle analyses by XRD, TGA and zeta potential demonstrated that the entire reaction process (1 h) can be divided into two stages: intercalation–oxidation (IO) and oxidation–exfoliation (OE; Fig. 3)^37^.

**Figure 4:**
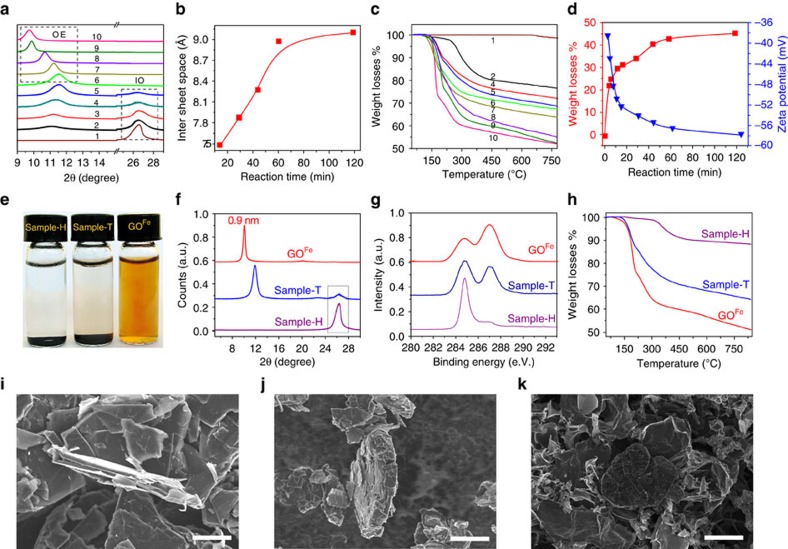
**Kinetics of the synthesis of GO**^**Fe**^. (**a**) XRD spectra of the samples taken from the synthesis process at the reaction times=0 min, 3 min, 5 min, 8 min, 11 min, 15 min 30 min, 45 min, 1 h and 2 h (**1**–**10**), respectively. (**b**) Interlayer spacing of selected samples at the OE stage versus reaction time. (**c**) TGA plots of the same samples as shown in **a**. (**d**) Weight loss of GO^Fe^ at 600 °C (left, red) and corresponding zeta potential (right, blue) as a function of reaction time. The kinetics of GO^Fe^ confirms that the whole reaction process completes in 1 h, including ~15 min of intercalation–oxidation and 45 min of oxidization–exfoliation. (**e**) Sample-H, Sample-T and GO^Fe^ (2 mg ml^−1^) placed in water, indicating that only GO^Fe^ is well-soluble. (**f**) XRD spectra, (**g**) C1s XPS spectra and (**h**) TGA plots of GO^Fe^, Sample-H and Sample-T with the reaction time 1 h. (**i**–**k**) SEM images of graphite, Sample-T and Sample-H, showing that the conventional Hummers methods with the oxidant of KMnO_4_ can only result in thick graphite-like particles rather than slGO in 1 h of reaction time. Scale bar, 20 μm (**i**–**k**).

In the first IO stage, concentrated sulphuric acid and K_2_FeO_4_ intercalate into the interlayer spacing of graphite. The oxidant then breaks the π–π conjugated structures at the edges and defects of the graphite, weakening the conjugate forces between pristine graphitic lamellae. In the corresponding XRD patterns (Fig. 4a), the appearance of a new peak at 2*θ*=11.4° accompanies the gradual fading of the 002 peak at 2*θ*=26.5° with increasing reaction time. At ~15 min, the diffraction peak of graphite at 26.5° disappears completely, indicating the completion of the IO stage and the formation of intercalated and partially oxidized graphite (GIO). An increase of the *d*-spacing of GIO is observed from 0.34 to 0.75 nm due to intercalation and oxidation. Intercalation and oxidation of graphite occur simultaneously, as confirmed by the dramatic mass loss from 0 to 30 wt % at 15 min in the corresponding TGA curves (Fig. 4c,d). The zeta potential also decreases rapidly to −52 mV (Fig. 4d), demonstrating the generation of negatively charged functional groups.

In the following OE stage, the oxidant further oxidized the carbon basal planes of GIO, giving rise to more functional groups and enlarging the *d*-spacing from 0.75 to 0.91 nm (Fig. 4a,b). In the TGA curves, the weight loss at 600 °C further increases from 30% at 15 min to 43% at 60 min (Fig. 4c). Notably, 100% slGO was achieved by 1 h, verifying the ultrafast OE process of our protocol. In fact, further extending the reaction time to 2 h gave little changes in the *d*-spacing, weight loss or zeta potential.

The entire reaction process is proposed by the following two steps:


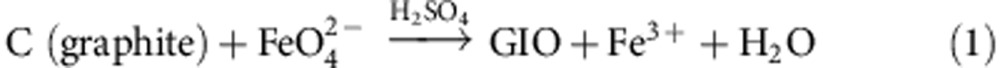



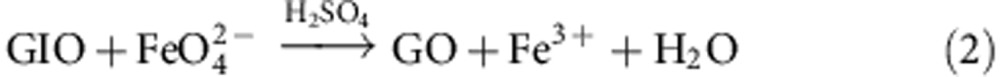


In addition, FeO_4_^2−^ reacts with H^+^ or water to produce atomic oxygen [O] that also effectively oxidizes carbon^38^. FeO_4_^2−^ and [O] work synergistically to efficiently yield slGO. The residual [O] forms oxygen gas, making both intercalation and exfoliation much more powerful and ultrafast^39^. Accordingly, all the reactions can be listed as follows:


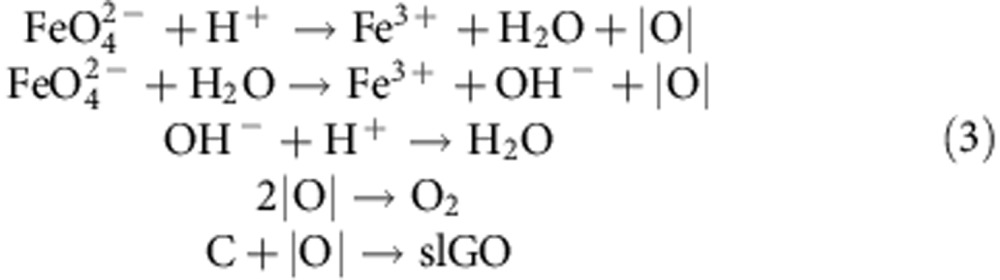


This unique reaction mechanism results in an ultrafast oxidation and exfoliation rates, providing slGO without additional ultrasonic treatment.

To analyze the oxidation efficiency of K_2_FeO_4_, we quantified the oxygen yield during GO^Fe^ production (Supplementary Methods). The results show that 70.2% of the K_2_FeO_4_ is consumed in the oxidation of graphite, 17.3% is decomposed into oxygen and 12.5% remains in the reaction suspension. This indicates that ~80% of the reacted K_2_FeO_4_ is converted into the oxygen-containing moieties of GO, confirming the extremely high oxidation efficiency of K_2_FeO_4_.

For comparison, we also studied samples oxidized for 1 h by two popular modified Hummers methods: Tour’s method^14^ (Sample-T) and Hirata’s method^13^ (Sample-H). Figure 4e shows that the two samples precipitated completely after 1 h of sonication after storage for 12 h, showing almost no solubility. XRD profile of Sample-H shows a strong graphite peak at 2*θ*=26.5° without the characteristic peak of graphite oxide. The Sample-T exhibits an obvious graphite peak and a graphite oxide peak at 2*θ*=11.9° (Fig. 4f), indicating strong oxidation but poor exfoliation. The XPS spectra of Sample-T and Sample-H reveal C/O ratios of 7.3 and 18.5, which are much higher than those found in GO^Fe^ (2.2; Fig. 4g). From the TGA plots of the two samples, ~32 and 10% mass losses are found, which are much lower than those found in GO^Fe^ (45%; Fig. 4h). A sample oxidized by KClO_3_ (Sample-B) for 1 h was also prepared and tested, indicating no solubility in water, a small degree of oxidation (C/O=13.4, 25 % wt loss) and poor exfoliation (with a strong graphite peak at 2*θ*=26.5° and graphite oxide peak at 2*θ*=12.5^o^; Supplementary Fig. 5). SEM images show that Sample-H has a similar thickness (~0.6 μm) as that of raw graphite and that a portion of Sample-T has a similar appearance to that of raw graphite, which confirm their multilayered state (Fig. 4i–k). These results demonstrate that our K_2_FeO_4_-based methodology, capable of both highly efficient oxidation and ultrafast exfoliation, is superior to the conventional methods.

Table 1 lists the comprehensive comparison of our K_2_FeO_4_-based methodology with the conventional methods. Generally, the new method possesses the following merits: ultrafast reaction rate, safe and environmentally friendly processing, no heavy metal pollution and ultralow cost. In our new method, 1 h is sufficient to obtain slGO without any additional post treatments such as ultrasonication or H_2_O_2_ washing, which are normally required in the Hummers methods. By comparison, the conventional methods require ~6 h–5 days of reaction time, as described in the 57 most cited studies on GO preparation (Supplementary Table 1). All of the conventional methods based on the KClO_3_ and KMnO_4_ oxidants as well as their optimized modifications produce toxic gases (ClO_2_, NO_*X*_) and explosive intermediates (for example, Mn_2_O_7_). In addition, for the KMnO_4_-based methodology, consumption of 1 ton of graphite would result in 1–5 ton of neat Mn^2+^ and 40–120 ton of sulphuric acid waste, leading to pollution, tedious post treatments and high costs. The high concentration of manganese in the system also stains GO with a Mn content of up to 97 p.p.m., which may cause significant injury to the body in cases where GO is used as a vehicle for drugs^40^,^41^. On the contrary, our K_2_FeO_4_-based approach has no safety or pollution issues, and the Mn content in GO^Fe^ is negligible (~0.025 p.p.m.). Moreover, the GO^Fe^ contains almost no iron (0.13 p.p.m.) despite the use of an iron-based oxidant, to the benefit of the eventual applications of GO and CCG.

Even though the fabrication is ultrafast at room temperature, the resulting GO^Fe^ is highly soluble in water and polar organic solvents and has both a composition and morphology comparable to GO^Mn^. As such, GO^Fe^ can be directly used in fields where GO^Mn^ has been demonstrated to be effective.

**Figure 5:**
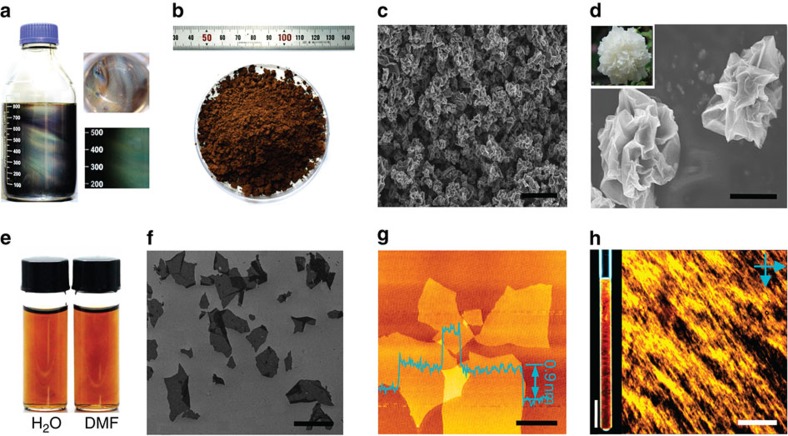
**Spray-dried GO**^**Fe**^ **powder for re-dissolving.** (**a**) Fresh GO^Fe^ LC solution of H_2_O with a concentration of 6 mg ml^−1^. (**b**) Macroscopic photograph of spray-dried GO^Fe^ powders with a density of 224 mg cm^−3^. (**c**,**d**) SEM images of GO^Fe^ powders, showing that the GO^Fe^ individual particles have a peony-like morphology. The insert of **d** is a peony. (**e**) Re-dissolved GO^Fe^ solutions of H_2_O and N,N-dimethylformamide with a concentration of 4 mg ml^−1^. (**f**) SEM image of re-dissolved single-layered GO^Fe^ sheets on Si/SiO_2_ substrate. (**g**) Tapping mode AFM image and height profile of re-dissolved GO^Fe^. (**h**) POM images of re-dissolved GO^Fe^ aqueous LCs in a quartz tube and a planar cell between crossed polarisers at a concentration of 4 mg ml^−1^. Scale bars, 3 μm (**c**), 500 nm (**d**), 10 μm (**f**), 2 μm (**g**) and 5 mm (**h**, left), 1 μm (**h**, right).

The preparation of GO powders is another very important issue that greatly affects the practical use of GO and its transport. Freeze-drying is commonly used to obtain solid GO. As shown in Supplementary Fig. 6, commercial GO powders apparently precipitate in minutes at 2 mg ml^−1^ even after 12 h of ultrasonic agitation. GO sheets laminate together as a result of π–π conjugation in the process of solvent removal. These aggregates are difficult to disrupt by the re-addition of solvents. We adopt a spray-drying method to control the morphology of the GO^Fe^ sheets and obtain only soluble GO powders (Fig. 5b). The dried GO powders can be completely dissolved in water and N,N-dimethylformamide (Fig. 5e) to form lyotropic LCs (Fig. 5h), identical to the fresh GO solutions before drying (Fig. 1b,h). The GO sheets are all dispersed in a single-layered state, as confirmed by SEM and AFM measurements (Fig. 5f,g).

As shown in Fig. 5c,d, the surfaces of the dried GO sub-microspheres are full of folds because the GO sheets shrink inwardly, forming peony-like 3D crumpled structures under the surface tension experienced in the spray-drying process. Such 3D crumpled sub-microsphere morphologies effectively prevent GO stacking, favouring the unfolding of sub-microspheres into plane sheet morphologies when re-dissolved in solvents. The GO^Fe^ sub-microsphere powder has a specific surface area of 1,467 m^2^ g^−1^, indicating 1–2 atomic layer structures (Supplementary Fig. 7). The dried GO^Fe^ powders are highly soluble in water and polar organic solvents. Significantly, our GO^Fe^ powder has a very high density (>224 mg cm^−3^), which facilitates its storage, transport and application. By comparison, despite a very low density (<30 mg cm^−3^) resulting from the freeze-drying process, the undissolved commercial GO powders have a very low specific surface area (<10 m^2^ g^−1^) due to their multilayer structure (Supplementary Fig. 8).

**Figure 6:**
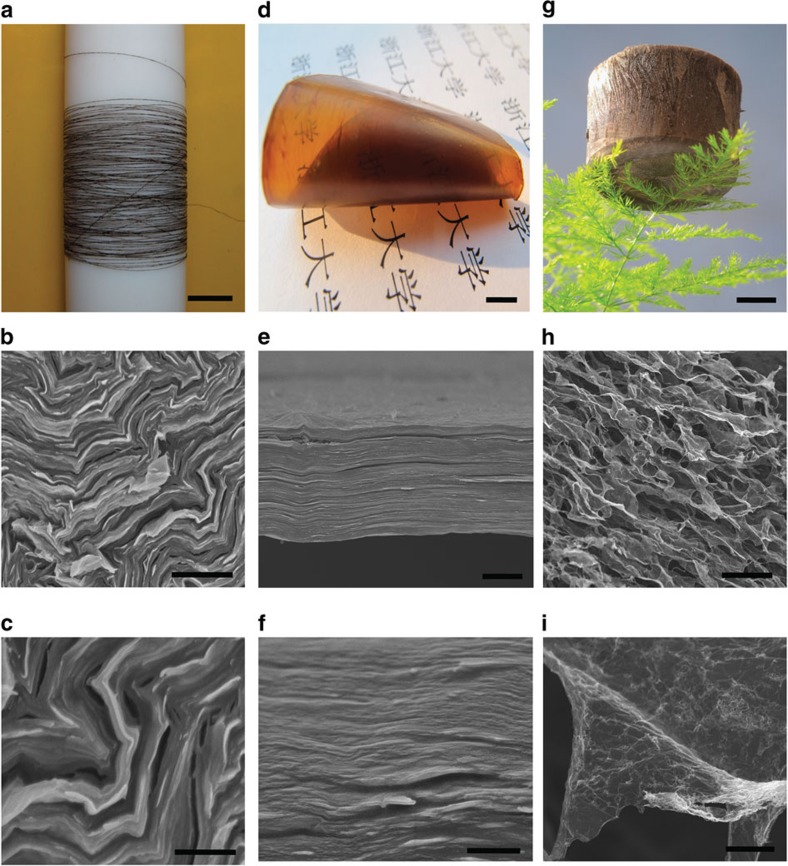
**Macroscopic assembled materials of re-dissolved GO**^**Fe**^. (**a**–**c**) A wet-spun 14-m long continuous fibre with diameter 10 μm and its SEM images at the cross-section of fibre. (**d**–**f**) A film made by the filtration method and its SEM image of a section. (**g**–**i**) Ultralight weight GO^Fe^ aerogel with a density of 2 mg cm^−3^ and its SEM images showing CNT-coated graphene morphology. Scale bars, 3 cm (**a**), 1 μm (**b**), 500 nm (**c**), 1 cm (**d**), 3 μm (**e**), 400 nm (**f**), 2 cm (**g**), 30 μm (**h**) and 2 μm (**i**).

The excellent dispersibility of the GO^Fe^ powders gives them superior solution processability, which is important in the fabrication of macroscopic materials (for example, 1D fibres, 2D films and 3D frameworks). A re-dissolved aqueous GO^Fe^ solution (~6 mg ml^−1^) shows a colourful optical texture typical of a nematic LC mesophase, identical to the appearance of fresh aqueous GO^Fe^ solutions (Fig. 5a and Supplementary Fig. 9)^36^. In a macroscopic quartz tube, the birefringence Schlieren texture between crossed polarisers can be seen with the naked eye across the entire solution (Fig. 5h). Such a LC suspension establishes the foundation to fabricate GO fibres, which has been demonstrated by our group and other independent researchers^22^,^42^. We subsequently obtained a continuous fibre by wet-spinning of the LC dope (Fig. 6a–c and Supplementary Fig. 10). It shows a highly compact and ordered structure, similar to previous GO fibres made directly from undried GO suspensions.

A film was made from the re-dissolved GO^Fe^ solution (Fig. 6d–f and Supplementary Fig. 11a) by the vacuum-assisted filtration method, which shows a well-aligned lamellar structure and comparable mechanical performance to GO^Mn^ papers^43^. After reduction with HI, our graphene film exhibits an electrical conductivity of 374 S cm^−1^ (Supplementary Fig. 11c), close to that (400 S cm^−1^) of defect-free graphene made by a high-shear exfoliation method^19^. A 3D aerogel prepared by a synergistic assembly of GO^Fe^ and carbon nanotubes (CNTs, 50 wt %) shows the same appearance and internal structure (Fig. 6g–i and Supplementary Fig. 12a) as an assembly prepared from GO^Mn^ and CNTs reported by our group previously^25^. After reduction with N_2_H_4_, an aerogel with a density of 2.0 mg cm^−3^ shows complete recovery even after 1,000 cycles of 87% compression. Significantly, the aerogel still remains elastic and intact after being compressed by a weight 5,000 times its own (Supplementary Fig. 12b). These results demonstrate the ‘true’ solution state of our re-dissolved GO^Fe^ and suggest the wide application of GO and CCG.

In conclusion, we established an industrially viable one-pot method for the production of slGO in 1 h at room temperature with ultralow cost based on the use of the novel oxidant of K_2_FeO_4_. The reaction process includes ~15 min of intercalation–oxidation and ~45 min of oxidization–exfoliation. The excellent oxidation capabilities of both K_2_FeO_4_ and the *in situ* generated atomic oxygen, accompanied by the exfoliation capacity of oxygen gas, make the intercalation, oxidation and exfoliation extremely powerful and ultrafast. The as-prepared slGO has a similar composition, chemical structure and solubility to materials prepared by the conventional Hummers method. Significantly, our dried slGO powders maintain excellent solubility in water and polar organic solvents and readily form stable LCs. Therefore, they retain the capacity to assemble into macroscopic materials such as continuous fibres, films and aerogels displayed by fresh GO solutions. The sulphuric acid solvent can be recycled in our protocol due to the ultrastrong oxidation capability of K_2_FeO_4_, which dramatically reduces the effluent and lowers the cost of GO. Our fast, eco-friendly and safe K_2_FeO_4_-based methodology circumvents the intrinsic problems associated with the prevailing methods of GO production, and it is easily amenable to the scalable production and industrial application of GO and CCG.

## Methods

### Synthesis of GO^Fe^

K_2_FeO_4_ (60 g, 6 wt equiv.) was added to concentrated H_2_SO_4_ (93%, 400 ml) at room temperature. Graphite (10 g, 1 wt equiv., 40 μm) was then added and the mixture was kept at room temperature for 1 h (note: the flask was not sealed due to the release of oxygen during the reaction). The mixture was centrifuged (10,000 r.p.m. for 3 min) to recycle the concentrated sulphuric acid. The paste-like product was collected by repeated centrifugation and washing with 1 l of water until the pH of the supernatant solution approached 7.

### Apparatus for characterizations

AFM images of GO sheets were taken in the tapping mode on a Nano Scope IIIA, with samples prepared by spin-coating diluted aqueous solutions onto freshly exfoliated mica substrates at 1,000 r.p.m.. SEM images were taken on a Hitachi S4800 field-emission SEM system. TEM was performed on a JEM-1200EX with an accelerating voltage of 120 kV. Zeta potential measurements were performed on a ZET-3000HS apparatus. Fourier transform infrared spectra were recorded on a PE Paragon 1000 spectrometer (film or KBr disk). Ultraviolet–visible spectra were obtained using a Varian Cary 300 Bio UV-visible spectrophotometer. Tensile tests were carried out on a HS-3200C at a loading rate of 1 mm min^−1^. XPS was performed using a PHI 5000C ESCA system operated at 14.0 kV. All binding energies were referenced to the C1s neutral carbon peak at 284.8 eV. TGA was carried out using a thermogravimetric analyser (PerkinElmer Pyris 1) from room temperature to 850 °C at 10 °C min^−1^ heating rate under air atmosphere. XRD data were collected with an X’Pert Pro (PANalytical) diffractometer using monochromatic Cu Kα1 radiation (*λ*=1.5406 Å) at 40 kV. Raman spectra were recorded on a Labram HRUV spectrometer operating at 632.8 nm. Mechanical property tests were carried out on a HS-3002C at a loading rate of 10% per minute. Elemental analyses were performed using an Agilent model 7700 × ICP-MS. BET surface area measurements were performed by nitrogen adsorption on a Quantachrome NOVA 2000 surface analyzer. POM observations were performed with a Nikon E600POL, and the liquid samples were loaded into the planar cells for observations. Combustion analysis was performed on an elemental analyzer (Vario Micro).

## Author contributions

C.G., L.P. and Z.X. conceived and designed the research; L.P. conducted the experiments and analyzed the data; Z. Liu, Z. Li, H.S., X.Z. and Y.W. discussed the data and provided some useful suggestions; C.G. supervised and directed the project; all of the authors read and revised the paper.

## Additional information

**How to cite this article:** Peng, L. *et al*. An iron-based green approach to 1-h production of single-layer graphene oxide. *Nat. Commun.* 6:5716 doi: 10.1038/ncomms6716 (2015).

## Supplementary Material

Supplementary Information Supplementary Figures 1-12, Supplementary Tables 1, Supplementary Methods and Supplementary References

## Figures and Tables

**Table 1:** A comparison of our K_2_FeO_4_-based methodology with KClO_3_- and KMnO_4_-based methodologies.

**Method (Year)**	**KClO**_**3**_ **based**			**KMnO**_**4**_ **based**				**K**_**2**_**FeO**_**4**_ **based**
	**Brodie**^8^	**Staudenmaier**^9^	**Hofmann**^10^	**Hummers**^11^	**Modified-1 (1999)**^12^	**Modified-2 (2004)**^13^	**Modified-3 (2010)**^14^	**Our work (2014)**
Reaction time	10 h	1–10 days	4 days	2–10 h	8 h	5 days	12 h	1 h
Interlayer spacing	5.95 Å	6.23 Å	—	6.67 Å	6.9 Å	8.3 Å	9.3 Å	9.0 Å
C/O ratio	2.16	—	—	2.25	2.3	1.8	—	2.2
Toxic gas	ClO_2_	ClO_2_, NO_*X*_	ClO_2_, NO_*X*_	NO_*X*_	—	NO_*X*_	—	No
Exploder	KClO_3_	KClO_3_	KClO_3_	Mn_2_O_7_	Mn_2_O_7_	Mn_2_O_7_	Mn_2_O_7_	No
Heavy metal in GO (p.p.m.)	—	—	—	97 (Mn^2+^)	—	—	87 (Mn^2+^)	0.025 (Mn^2+^)0.13 (Fe^3+^)
Mn^2+^ generated (for 1 ton graphite)	—	—	—	1 ton	1 ton	1.5 ton	2 ton	0

C, carbon; GO, graphene oxide; O, oxygen.
