# Supplementary material for: An iron-based green approach to 1-h production of single-layer graphene oxide
Source: Nat Commun. 2015 Jan 21;6:5716. doi: 10.1038/ncomms6716 (PMC4354147; doi:10.1038/ncomms6716)
Supplement: Supplementary Information — Supplementary Figures 1-12, Supplementary Tables 1, Supplementary Methods and Supplementary References [file ncomms6716-s1.pdf]

Supplementary Figures

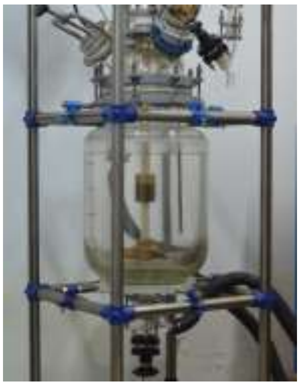

Supplementary Figure 1 | Picture of reactor (20 L) for the scalable preparation of GO^{Fe}.

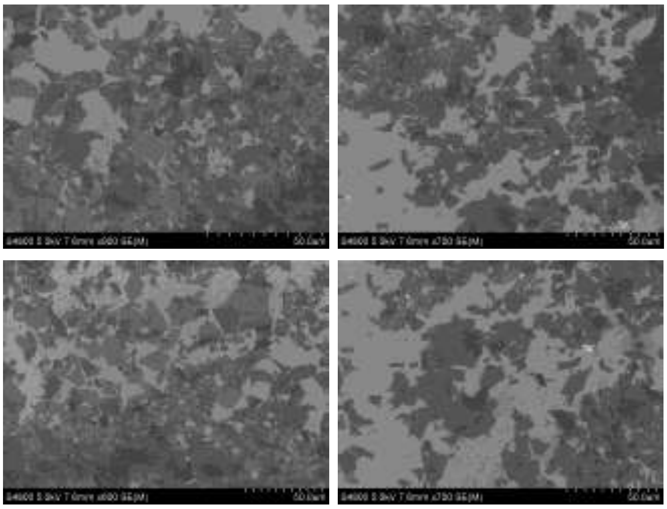

Supplementary Figure 2 | SEM images of GO^{Fe} sheets.

a b

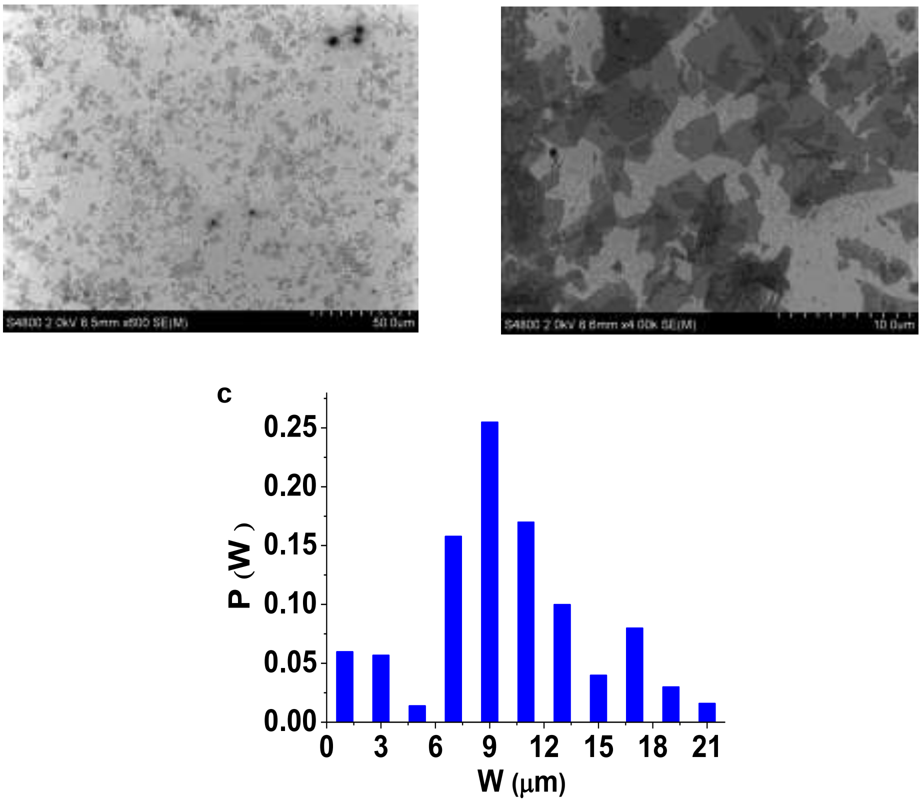

Supplementary Figure 3 | (**a, b**) SEM images of GO^{Mn} on Si/SiO_2 substrate. (**c**) Size distribution of GO^{Mn}, counted and calculated from (**a, b**).

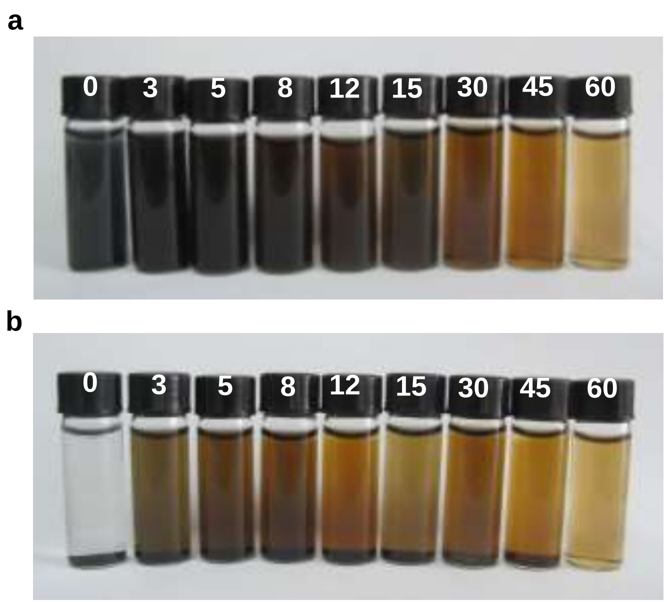

Supplementary Figure 4 | (**a**) Graphite oxide dispersions after 0 min, 3 min, 5 min, 8 min, 12 min, 15 min, 30 min, 45 min and 60 min reaction with a concentration of 0.5 mg mL^{-1}. (**b**) Counterparts of (**a**) after staying for 24 h.

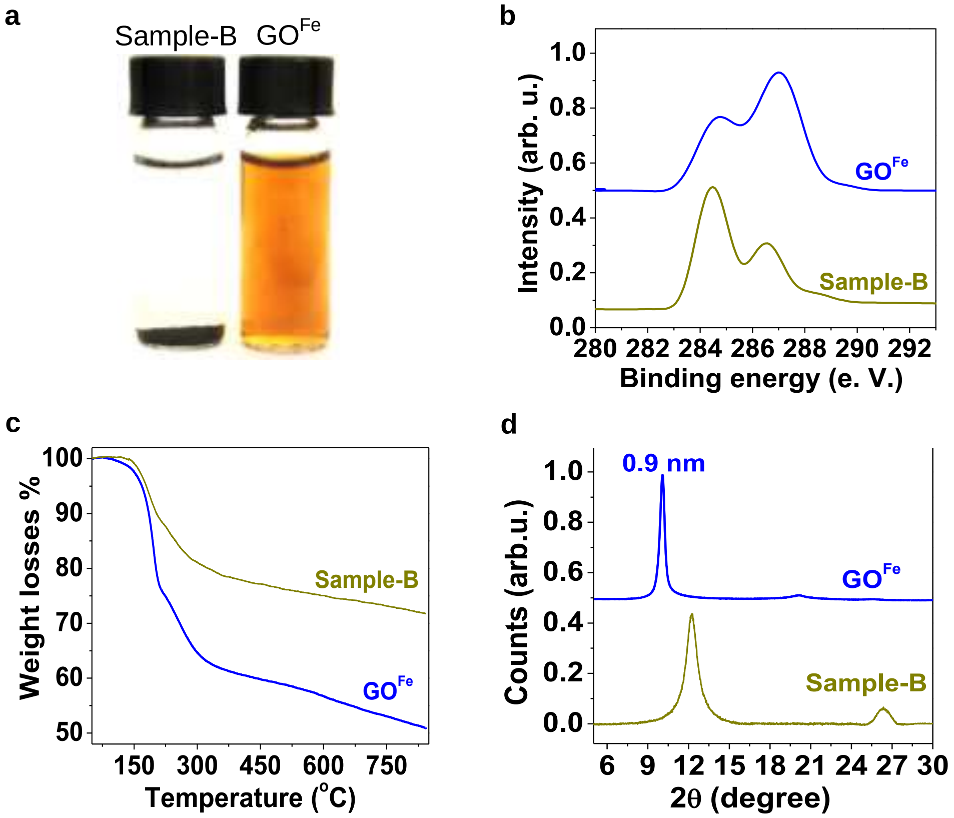

Supplementary Figure 5 | Comparison of GO^{Fe} and Sample-B ($KClO_3$ oxidant for 1 h reaction). (**a**) Photograph of GO^{Fe} and Sample-B placed in water after staying for 2 h. (**b**) C1s XPS spectra, (**c**) TGA plots, and (**d**) XRD spectra of GO^{Fe} and Sample-B.

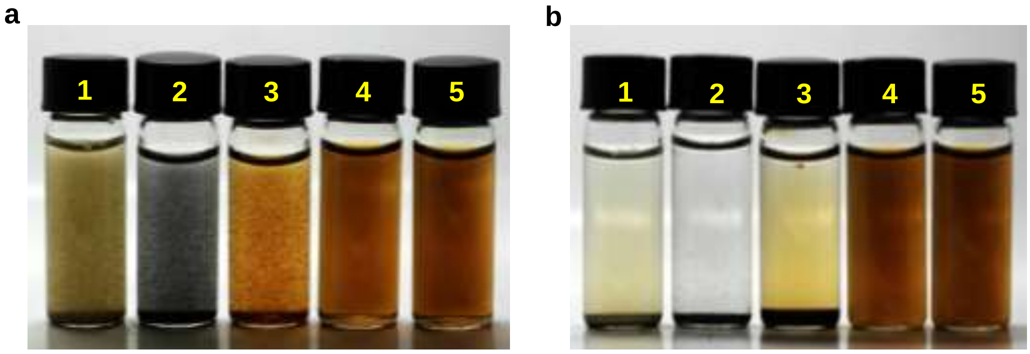

Supplementary Figure 6 | (**a**) Re-dispersed GO in water from commercial dried GO powders (**1**, **2**, **3**) after ultrasound for 12 h, our spray-dried GO^{Fe} powders (**4**) and the fresh GO^{Fe} solution (**5**). (**b**) Counterparts of (**a**) after staying for 20 minutes.

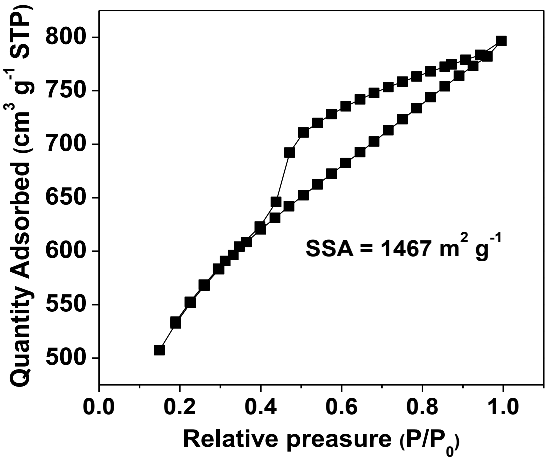

Supplementary Figure 7 | Nitrogen adsorption/desorption isotherm for the GOFe powders.

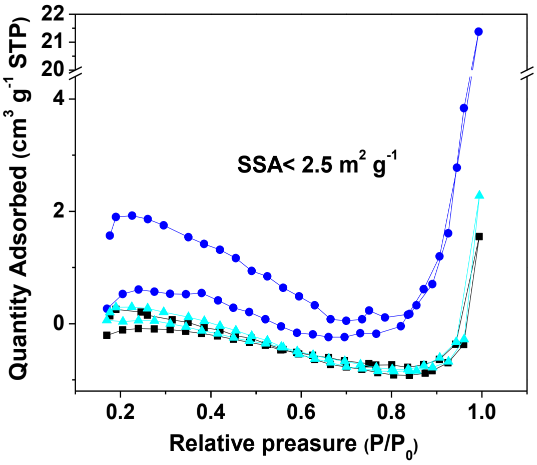

Supplementary Figure 8 | Nitrogen adsorption/desorption isotherm for three commercial dried GO powders.

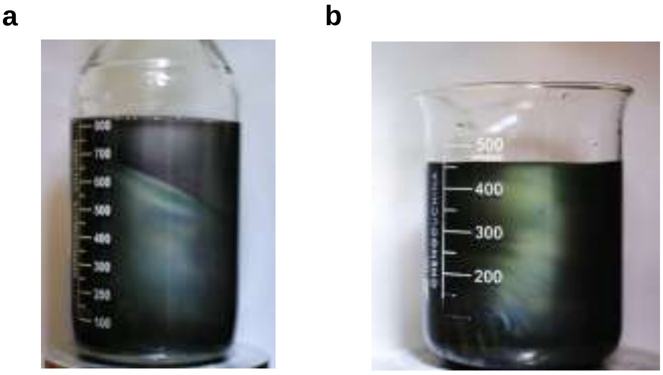

Supplementary Figure 9 | (**a**) GO^{Fe} solution and (**b**) re-dissolved GO^{Fe} solution with a concentration of 6 mg mL^{-1}, indicating macroscopic liquid crystalline fluid for the whole solution.

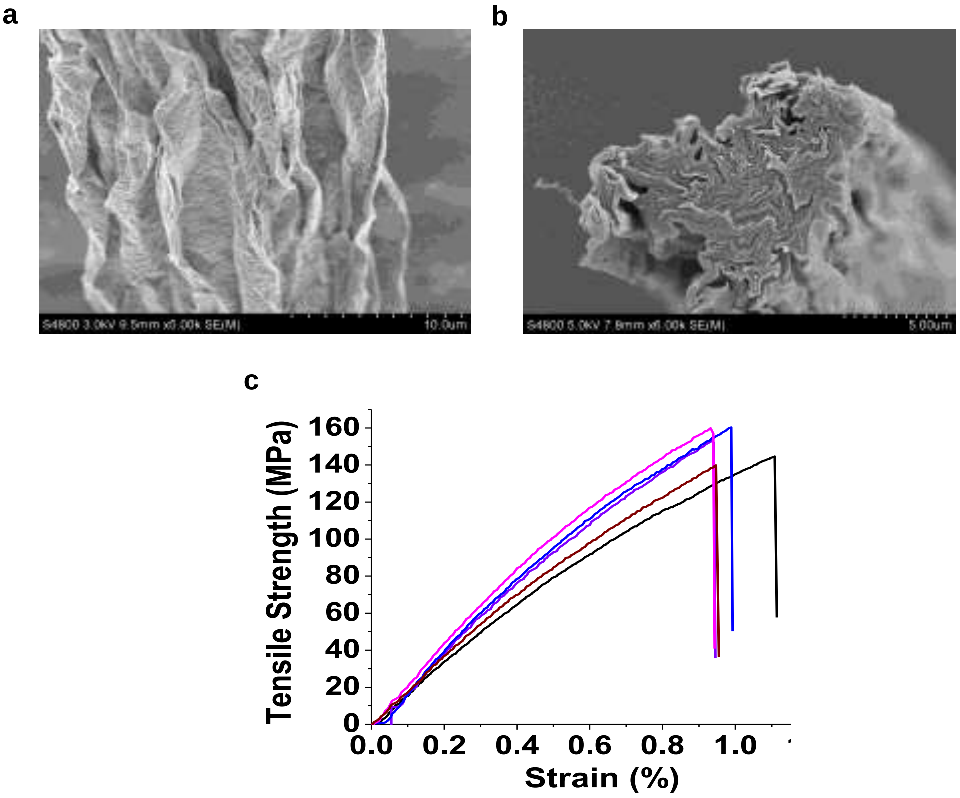

Supplementary Figure 10 | (**a, b**) SEM images of GO^{Fe} fibre and (**c**) its strength-strain curve.

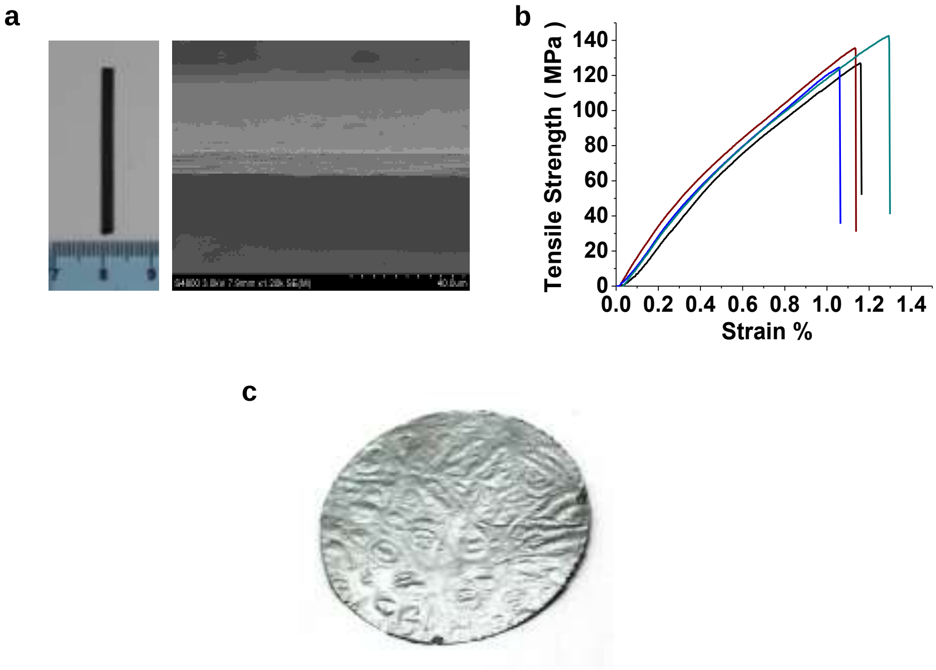

Supplementary Figure 11 | (**a**) The sample of GO^{Fe} film for the tensile tests. (**b**) Strength-strain curve of GO^{Fe} film. (**c**) Silver-like GO^{Fe} film reduced by HI.

a

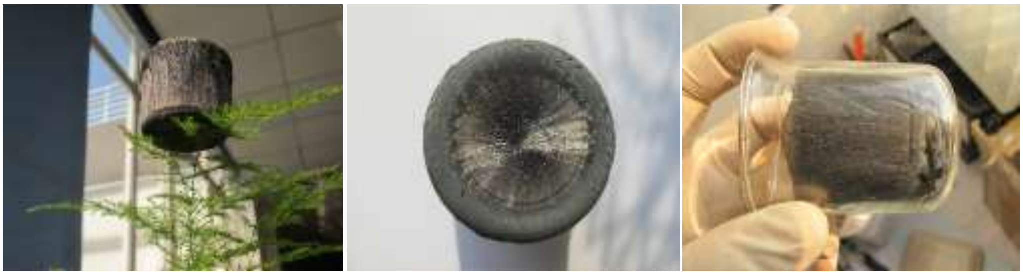

b

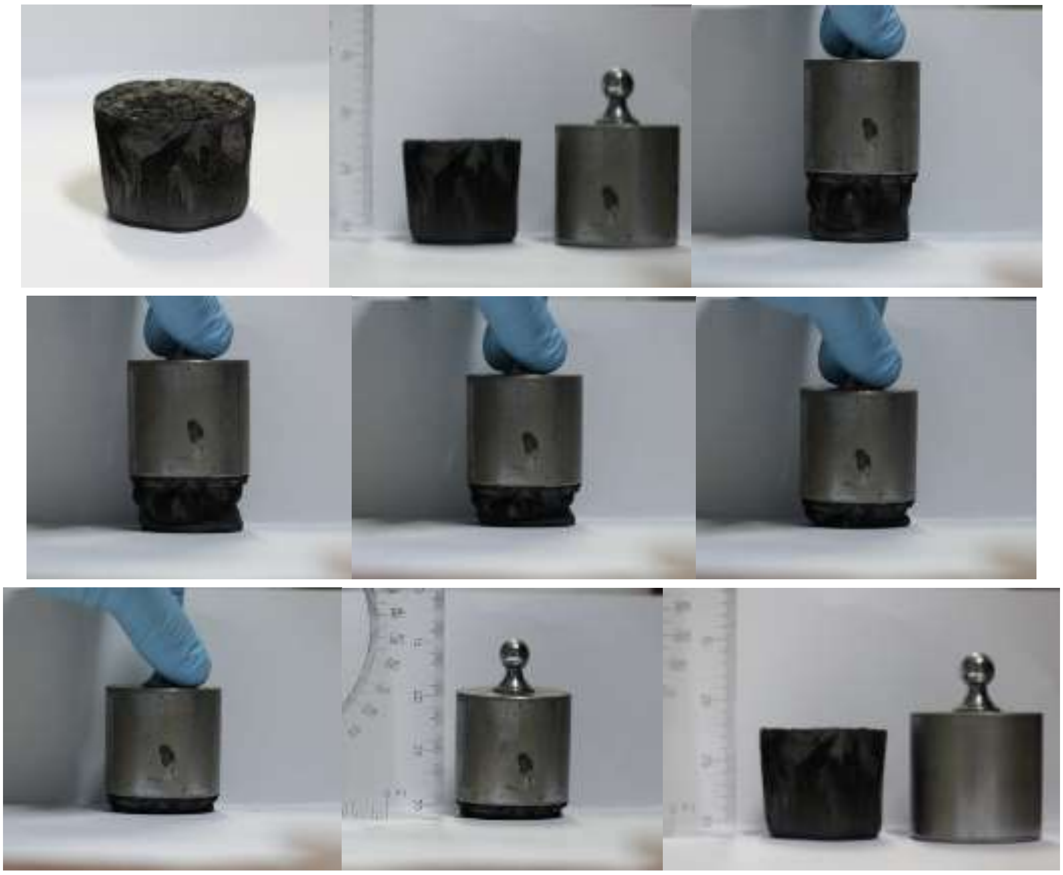

Supplementary Figure 12 | (**a**) Aerogel without reduction prepared by freeze-drying aqueous solution of CNTs and GO^{Fe} sheets. (**b**) GO^{Fe} aerogel reduced by N_2H_4 and the demonstration of its excellent elasticity after compression.

Supplementary Tables

Ref.	Method	Size of graphite	Preparation steps (Sonication)	Reaction temperature	Ingredients for 1g graphite	Reaction time
Our work	K_2FeO_4-based method	40 μm (1g)	1	r. t.	40 mL H_2SO_4<br>6 g K_2FeO_4	1h
1[1]	Staudenmaier method	45 μm (1 g)	1	r. t.	17.5 mL H_2SO_4<br>9 mL $fHNO_3$<br>11 g $KClO_3$	>96 h
2[2]	Staudenmaier method	45 μm (1 g)	1	r. t.	17.5 mL H_2SO_4<br>9 mL HNO_3<br>11 g $KClO_3$	>96 h
3[3]	modified Hummers method	4 μm (1 g)	1 (Y)	98 °C	82 mL H_2SO_4<br>0.75 g $NaNO_3$<br>4.5 g $KMnO_4$<br>3 mL H_2O_2	>120 h
4[4]	modified Hummer's method	1 g	1	105 ºC	50 g NaCl<br>23 mL H_2SO_4<br>6 g $KMnO_4$<br>10 mL H_2O_2	>14.5 h
5[5]	modified Hummers method	45 μm (12 g)	2	80 ºC	44 mL H_2SO_4<br>0.83 g $K_2S_2O_8$<br>0.83 g P_2O_5<br>5 g $KMnO_4$<br>4.2 mL H_2O_2	>6.5 h
6[6]	modified Hummers method	150 μm (3 g)	1	50 °C	120 mL H_2SO_4<br>13.3 mL H_3PO_4<br>6 g $KMnO_4$<br>1 mL H_2O_2	>12 h

7[7]	modified Hummers method	250 µm (1 g)	1	r. t.	17.5 mL H_2SO_4<br>9 mL HNO_3<br>11 g $KClO_3$	>120 h
8[8]	modified Hummers and Offeman's method	45 µm (3 g)	1 (Y)	80 °C	40 mL H_2SO_4<br>8.3 g $K_2S_2O_8$<br>8.3 g P_2O_5	>6 h
9[9]	—	5 g	1 (Y)	40 °C	18 mL H_2SO_4<br>6 mL HNO_3	>96 h
10[10]	modified Hummers method	49 µm (5 g)	1	98 °C	0.5 g $NaNO_3$<br>24 mL H_2SO_4<br>3 g $KMnO_4$<br>10 mL H_2O_2	>32 h
11[11]	modified Hummers method	45 µm (20 g)	1	35 °C	24.5 mL H_2SO_4<br>0.5 g $K_2S_2O_8$<br>0.5 g P_2O_5<br>3 g $KMnO_4$<br>2.5 mL H_2O_2	>16 h
12[12]	modified Brodie method	74 µm (1 g)	1 (Y)	—	8.5 g $NaClO_3$<br>20 mL $fHNO_3$	>24 h
13[13]	modified Hummers method	5 g	1	—	0.5 g $NaNO_3$<br>13 mL H_2SO_4<br>3 g $KMnO_4$<br>10 mL H_2O_2	>8 h
14[14]	modified Hummers method	1 g	1 (Y)	40 °C	20 g NaCl<br>0.1 g $NaNO_3$<br>23 mL H_2SO_4<br>0.5 g $KMnO_4$<br>10 mL H_2O_2	>26 h
15[15]	Hummers method	4 g	1 (Y)	r. t.	29 mL H_2SO_4<br>2 g $K_2S_2O_8$<br>2 g P_2O_5<br>3 g $KMnO_4$<br>0.5 g $NaNO_3$<br>2.5 mL H_2O_2	>8 h

16[16]	modified Hummers method	>150 μm (15 g)	1 (Y)	r. t.	0.5 g $NaNO_3$<br>23.3 mL H_2SO_4<br>3 g $KMnO_4$<br>10 mL H_2O_2	21 h
17[17]	Staudenmaier method	5 g	1	r. t.	17.5 mL H_2SO_4<br>95 mL $fHNO_3$<br>11 g $KClO_3$	>96 h
18[18]	Hummers method	45 μm (4 g)	1	80 °C	82.5 mL H_2SO_4<br>1.5 g $K_2S_2O_8$<br>1.5 g P_2O_5<br>8.75 g $KMnO_4$<br>25 mL H_2O_2	>10 h
19[19]	modified Hummers method	1 g	1	40 °C	50 g NaCl<br>23 mL H_2SO_4<br>0.75 g $KMnO_4$<br>10 mL H_2O_2<br>0.1 g $NaNO_3$	>25 h
20[20]	modified Hummers method	4 μm (1 g)	1	98 °C	0.75 g $NaNO_3$<br>82 mL H_2SO_4<br>4.5 g $KMnO_4$<br>6 mL H_2O_2	>120 h
21[21]	modified Hummers method	12 g	1	80 °C	42.5 mL H_2SO_4<br>0.83 g $K_2S_2O_8$<br>0.83 g P_2O_5<br>5 g $KMnO_4$<br>4.2 mL H_2O_2	>10 h
22[22]	modified Hummers method	1 g	1 (Y)	80 °C	24.5 mL H_2SO_4<br>0.5 g $K_2S_2O_8$<br>0.5 g P_2O_5<br>3 g $KMnO_4$<br>2 mL H_2O_2	>8 h

23[23]	Staudenmaier method	5 g	1	r. t.	17.5 ml H_2SO_4<br>9 mL HNO_3<br>11 g $KClO_3$	>96 h
24[24]	Hummers method	45 µm<br>(20 g)	1	80 °C	24.5 mL H_2SO_4<br>0.5 g $K_2S_2O_8$<br>0.5 g P_2O_5<br>3 g $KMnO_4$<br>2.5 mL H_2O_2	>8 h
25[25]	Hummers method	45 µm<br>(2 g)	1	80 °C	6 mL H_2SO_4<br>1.5 g $K_2S_2O_8$<br>1.5 g P_2O_5<br>12.5 g $KMnO_4$<br>19.5 mL H_2O_2	>9 h
26[26]	modified Hummers method	1 g	1	98 °C	0.75 g $NaNO_3$<br>85 mL H_2SO_4<br>6 g $KMnO_4$<br>6 mL H_2O_2	>168 h
27[27]	modified Hummers method	4 µm<br>(5 g)	1	98 °C	0.75 g $NaNO_3$<br>82 mL H_2SO_4<br>4.5 g $KMnO_4$<br>6 mL H_2O_2	>120 h
28[28]	modified Hummers method	4 µm<br>(1 g)	1	98 °C	0.75 g $NaNO_3$<br>82 mL H_2SO_4<br>4.5 g $KMnO_4$<br>6 mL H_2O_2	>120 h
29[29]	modified Hummers method	0.3 g	1	80 °C	48 mL H_2SO_4<br>1.67 g $K_2S_2O_8$<br>1.67 g P_2O_5<br>5 g $KMnO_4$<br>6.7 mL H_2O_2	>8.5 h

30[30]	Hummers method	45 μm (20 g)	1	35 °C	24.5 mL H_2SO_4<br>0.5 g $K_2S_2O_8$<br>0.5 g P_2O_5<br>3 g $KMnO_4$<br>2.5 mL H_2O_2	>8 h
31[31]	modified Hummers method	1 g	1	70 °C	40 g NaCl<br>23 mL H_2SO_4<br>0.5 g $KMnO_4$<br>0.1 g $NaNO_3$<br>10 mL H_2O_2	>25 h
32[32]	modified Hummers method	45 μm (6 g)	1	90 °C	42.5 mL H_2SO_4<br>0.83 g $K_2S_2O_8$<br>0.83 g P_2O_5<br>5 g $KMnO_4$<br>4.2 mL H_2O_2	8.5 h
33[33]	modified Hummers method	2 g	1	35 °C	48 mL H_2SO_4<br>0.5 g $NaNO_3$<br>3 $KMnO_4$<br>2.5 mL H_2O_2	>18 h
34[34]	modified Hummers method	1 g	1	70 °C	23 mL H_2SO_4<br>0.1 g $NaNO_3$<br>3 g $KMnO_4$<br>10 mL H_2O_2<br>50 g NaCl	>24 h
35[35]	modified Hummers method	0.3 g	1	80 °C	48 mL H_2SO_4<br>1.67 g $K_2S_2O_8$<br>1.67 g P_2O_5<br>5 g $KMnO_4$<br>6.7 mL H_2O_2	8.5 h
36[36]	modified Hummers method	24 μm (10 g)	1	r. t.	62.1 g H_2SO_4<br>0.75 g $NaNO_3$<br>4.5 g $KMnO_4$<br>3 g H_2O_2	>120 h

37[37]	modified Hummers method	0.3 g	1	80 °C	48 mL H_2SO_4<br>1.67 g $K_2S_2O_8$<br>1.67 g P_2O_5<br>5 g $KMnO_4$<br>6.7 mL H_2O_2	8.5 h
38[38]	modified Hummers method	45 μm (5 g)	1	r. t.	36 mL H_2SO_4<br>12 mL fHNO_3<br>5 g $KMnO_4$<br>6 mL H_2O_2	>120 h
39[39]	modified Hummers method	0.2 g	1	r. t.	80 mL H_2SO_4<br>0.875 g $NaNO_3$<br>4.5 g $KMnO_4$<br>3 mL H_2O_2	>120 h
40[40]	Hummers method	0.15 g	1	80 °C	867 mL H_2SO_4<br>33.3 g $K_2S_2O_8$<br>33.3 g P_2O_5<br>400 g $KMnO_4$<br>333 mL H_2O_2	>9.5 h
41[41]	modified Hummers method	3 g	1	80 °C	44 mL H_2SO_4<br>0.83 g $K_2S_2O_8$<br>0.83 g P_2O_5<br>5 g $KMnO_4$<br>6.7 mL H_2O_2	6.5 h
42[42]	modified Hummers method	24 μm (10 g)	1	r. t.	62.1 g H_2SO_4<br>0.75 g $NaNO_3$<br>4.5 g $KMnO_4$<br>3 g H_2O_2	>120 h
43[43]	modified Hummers method	45 μm (3 g)	1	80 °C	44 mL H_2SO_4<br>0.83 g $K_2S_2O_8$<br>0.83 g P_2O_5<br>5 g $KMnO_4$<br>6.7 mL H_2O_2	8.5 h

44[44]	modified Hummers method	3000-5000 μm (2 g)	1 (Y)	80 °C	66 mL H_2SO_4<br>7.5 g $KMnO_4$<br>10 mL H_2O_2	8.5 h
45[45]	modified Hummers method	45 μm (3 g)	2	80 °C	44 mL H_2SO_4<br>0.83 g $K_2S_2O_8$<br>0.83 g P_2O_5<br>5 g $KMnO_4$<br>6.7 mL H_2O_2	>8.5 h
46[46]	modified Hummers method	1 g	1	98 °C	46 mL H_2SO_4<br>1 g NaNO3<br>6 g $KMnO_4$<br>20 mL H_2O_2	6.25 h
47[47]	modified Hummers method	1.5 g	1	90 °C	30 mL H_2SO_4<br>0.67 g $NaNO_3$<br>4 g $KMnO_4$<br>6.7 mL H_2O_2	>23 h
48[48]	modified Hummers method	5 g	1	r. t.	38.8 mL H_2SO_4<br>0.9 g KNO_3<br>4.5 g $KMnO_4$<br>3 g H_2O_2	>120 h
49[49]	modified Hummers method	5 g	1	r. t	38.8 mL H_2SO_4<br>0.76 g $NaNO_3$<br>4.5 g $KMnO_4$<br>3 g H_2O_2	>120 h
50[50]	modified Hummers method	< 20 μm (1 g)	1	80°C	98 mL H_2SO_4<br>2 g $K_2S_2O_8$<br>2 g P_2O_5<br>15 g $KMnO_4$<br>10 mL H_2O_2	8.25 h
51[51]	modified Hummers method	1.5 g	1	80°C	46.7 mL H_2SO_4<br>0.83 g $K_2S_2O_8$<br>0.83 g P_2O_5<br>5 g $KMnO_4$<br>6.7 mL H_2O_2	6.5 h

52[52]	modified Hummers method	4 g	1	80°C	29 mL H_2SO_4<br>2 g $K_2S_2O_8$<br>2 g P_2O_5<br>3 g $KMnO_4$<br>2.5 mL H_2O_2<br>0.5g NaNO3	>13 h
53[53]	modified Hummers method	0.3 g	1	80°C	48 mL H_2SO_4<br>1.67 g $K_2S_2O_8$<br>1.67 g P_2O_5<br>5 g $KMnO_4$<br>6.7 mL H_2O_2	8.5 h
54[54]	Hummers method	3 g	2	98°C	12.3 mL H_2SO_4<br>0.83 g $K_2S_2O_8$<br>0.83 g P_2O_5<br>1 g $KMnO_4$<br>0.17 g $NaNO_3$<br>1 mL H_2O_2	>60 h
55[55]	Hummers method	1 g	2	80°C	24.5 mL H_2SO_4<br>0.5 g $K_2S_2O_8$<br>0.5 g P_2O_5<br>3 g $KMnO_4$<br>2 mL H_2O_2	>8 h
56[56]	modified Hummers method	0.3 g	1	80°C	48 mL H_2SO_4<br>1.67 g $K_2S_2O_8$<br>1.67 g P_2O_5<br>5 g $KMnO_4$<br>6.7 mL H_2O_2	8.5 h
57[57]	modified Hummers method	3 g	1	80°C	44 mL H_2SO_4<br>0.83 g $K_2S_2O_8$<br>0.83 g P_2O_5<br>5 g $KMnO_4$<br>6.7 mL H_2O_2	8.5 h
The methods come from the most cited papers describing slGO obtained by the oxidation-exfoliation method.						

Supplementary Table 1 | Oxidation exfoliation methods to obtain slGO.

Supplementary Methods

Experimental materials.

Natural graphite flakes (40 μ m) were purchased from Qingdao Henglide Graphite Co., Ltd. K_2FeO_4 was obtained from Hubei CSW-China Chemistry Co., Ltd. All the other reagents were purchased from Sinopharm Chemical Reagent Co., Ltd. and used as received.

Scalable preparation of GO^{Fe}.

K_2FeO_4 (25 kg) was added into concentrated H_2SO_4 (93%, 16 L) slowly. Then graphite (500 g, 40 μm) was added slowly and the mixture was stirred for 1 h. The mixture was centrifuged (10000 rpm for 3min) to recycle the concentrated sulfuric acid. The paste-like product was collected by repeated centrifugation and water washing until the pH of the decantate approached 7, yielding 750 g of spray-dried powder.

Synthesis of GO^{Mn}.

The preparation process of GO^{Mn} was according to reference 24.

Synthesis of sample-T, sample-H, and sample-B.

The preparation processes of sample-T, sample-H, and sample-B were according to references of 14, 13 and 8.

Preparation of GO fibre, film and carbon aerogel.

The preparation process of GO fibres, films and carbon aerogels followed the references of 22-24 in the main text.

Measurement of O_2.

The mass of O_2 released from the reaction system of K_2FeO_4, H_2SO_4 and graphite was measured by the mass loss of the reaction system.

Typically, K_2FeO_4 (6 g) was added into concentrated H_2SO_4 (40 mL) to carry on the control experiment between K_2FeO_4 and H_2SO_4 in the absence of graphite until no gas releasing from the system. The mass loss before and after reaction is about 0.67 g, corresponding to the oxygen generated by the reaction between K_2FeO_4 and H_2SO_4.

In another experiment, graphite (1 g, 40 μm) and K_2FeO_4 (6 g) were added to concentrated H_2SO_4 (40 mL). The mixture was stirred for 1 h. In this process, 0.116 g

mass loss was measured, corresponding to the oxygen decomposed from K_2FeO_4. The suspension was poured slowly into 60 mL water and stirred for 2 h, and 0.084 g of mass loss was found, corresponding to the oxygen generated by the remained K_2FeO_4 after production of GO.

Supplementary References

1. Schniepp, H. C. *et al.* Functionalized single graphene sheets derived from splitting graphite oxide. *J. Phys. Chem.* **110**, 8535–8539 (2006).

2. McAllister, M. J. *et al.* Single sheet functionalized graphene by oxidation and thermal expansion of graphite. *Chem. Mater.***19**, 4396–4404 (2007)

3. Becerril, H. A. *et al.* Evaluation of solution-processed reduced graphene oxide filmsas transparent conductors. *ACS Nano* **2**, 463–470 (2008).

4. Liu, Z., Robinson, J. T., Sun, X. & Dai, H. Pegylated nanographene oxide for delivery of water-insoluble cancer drugs. *J. Am. Chem. Soc.* **130**, 10876–10877 (2008).

5. Gilje, S., Han, S., Wang, M., Wang, K. L. & Kaner, R. B. A chemical route to graphene for device applications. *Nano Lett.* **7**, 3394–3398 (2007).

6. Marcano, D. C. *et al.* Improved synthesis of graphene oxide. *ACS Nano* **4,** 4806–4814 (2010).

7. Wang, G. *et al.* Facile synthesis and characterization of graphene nanosheets. *J. Phys. Chem. C* **112**, 8192–8195 (2008).

8. Zhang, H., Lv, X., Li, Y., Wang, Y. & Li, J. P25-graphene composite as a high performance photocatalyst. *ACS Nano* **4,** 380–386 (2010).

9. Niyogi, S. *et al.* Solution properties of graphite and graphene. *J. Am. Chem. Soc.* **128**, 7720–7721 (2006).

10. Zhang, K., Zhang, L., Zhao, X. & Wu, J. Graphene/polyaniline nanofiber composites as supercapacitor electrodes. *Chem. Mater.* **22**, 1392-1401 (2010).

11. Kovtyukhova, N. *et al.* Layer-by-layer assembly of ultrathin composite films from micron-sized graphite oxide sheets and polycations. *Chem. Mater.* **11**, 771–778 (1999).

12. Shin, H. *et al.* Efficient reduction of graphite oxide by sodium borohydride and its effect on electrical conductance. *Adv. Funct. Mater.* **19**, 1987-1992 (2009).

13. Liu, C., Yu, Z., Neff, D., Zhamu, A. & Jang, B. Z. Graphene-based supercapacitor with an ultrahigh energy density. *Nano Lett.* **10**, 4863-4868 (2010).

14. Liang, Y. *et al.* Co_3O_4 nanocrystals on graphene as a synergistic catalyst for oxygen reduction reaction. *Nat. Mater.* **10**, 780-786 (2011).

15. He, S. *et al.* A graphene nanoprobe for rapid, sensitive, and multicolor fluorescent DNA analysis. *Adv. Funct. Mater*. **20**, 453-459 (2010).

16. Scheuermann, G. M., Rumi, L., Steurer, P., Bannwarth, W. & Mulhaupt, R. Palladium nanoparticles on graphite oxide and its functionalized graphene derivatives as highly active catalysts for the suzuki-miyaura coupling reaction. *J. Am. Chem. Soc.* **131**, 8262–8270 (2009).

17. Lomeda, J. R., Doyle, C. D., Kosynkin, D. V., Hwang, W. F. & Tour, J. M. Diazonium functionalization of surfactant-wrapped chemically converted graphene sheets. *J. Am. Chem. Soc.* **130**, 16201–16206 (2008).

18. Tang, L. *et al.* Preparation, structure, and electrochemical properties of reduced graphene sheet films. *Adv. Funct. Mater.* **19**, 2782-2789 (2009).

19. Li, X. *et al.* Simultaneous nitrogen doping and reduction of graphene oxide. *J. Am. Chem. Soc.* **131**, 15939–15944 (2009).

20. Liu, Z. et al. Organic photovoltaic devices based on a novel acceptor material: Graphene. *Adv. Mater.* **20**, 3924-3930 (2008).

21. Zhu, C., Guo, S., Fang, Y. & Dong, S. Reducing sugar: New functional molecules for the green synthesis of graphene nanosheets. *ACS Nano.* **4,** 2429–2437 (2010).

22. Zhang, L., Xia, J., Zhao, Q., Liu, L. & Zhang, Z. Functional graphene oxide as a nanocarrier for controlled loading and targeted delivery of mixed anticancer drugs. *Small* **6**, 537-544 (2010).

23. Rafiee, M. A. *et al.* Enhanced mechanical properties of nanocomposites at low graphene content. *ACS Nano* **3,** 3884–3890 (2009).

24. El-Kady, M. F., Strong, V., Dubin, S. & Kaner, R. B. Laser scribing of high-performance and flexible graphene-based electrochemical capacitors. *Science* **335**, 1326-1330 (2012).

25. Wang, Y. *et al.* Aptamer/graphene oxide nanocomplex for in situ molecular probing in living cells. *J. Am. Chem. Soc.* **132**, 9274–9276 (2010).

26. Yang, X. *et al.* Superparamagnetic graphene Oxide–Fe_3O_4 nanoparticles hybrid for controlled targeted drug carriers. *J. Mater. Chem.* **19**, 2710–2714 (2009).

27. Yang, N., Zhai, J., Wang, D., Chen, Y. & Jiang, L. Two-dimensional graphene bridges enhanced photoinduced charge transport in dye-sensitized solar cells. *ACS Nano* **4**, 887-894 (2010).

28. Vivekchand, S. R. C., Rout, C. S., Subrahmamyam, K. S., Govindaraj, A. & Rao, C. N. R. Graphene-based electrochemical supercapacitors. *J. Chem. Sci.* **120**, 9–13 (2008).

29. Zhou, X. *et al.* In situ synthesis of metal nanoparticles on single-layer graphene oxide and reduced graphene oxide surfaces. *J. Phys. Chem.* C **113**, 10842–10846 (2009).

30. Li, Y., Tang, L. & Li, J. Preparation and electrochemical performance for methanol oxidation of pt/graphene nanocomposites. *Electrochem. Commun.* **11**, 846–849 (2009).

31. Wang, H. *et al.* Graphene–wrapped sulfur particles as a rechargeable lithium-sulfur battery cathode material with high capacity and cycling stability. *Nano Lett.* **11**, 2644–2647 (2011).

32. Liu, J., Fu, S., Yuan, B., Li, Y. & Deng, Z. Toward a universal "adhesive nanosheet" For the assembly of multiple nanoparticles based on a protein-induced reduction/decoration of graphene oxide. *J. Am. Chem. Soc.* **132**, 7279–7281 (2010).

33. Zhu, X., Zhu, Y., Murali, S., Stoller, M. D. & Ruoff, R. S. Nanostructured reduced graphene Oxide/Fe_2O_3 composite as a high-performance anode material for lithium ion batteries. *ACS Nano* **5**, 3333-3338 (2011).

34. Wang, H., Robinson, J. T., Li, X. & Dai, H. Solvotherma lreduction of chemically exfoliated graphene sheets. *J. Am. Chem. Soc.* **131**, 9910–9911 (2009)

35. Wang, Z., Zhou, X., Zhang, J., Boey, F. & Zhang, H. Direct electrochemical reduction of single-layer graphene oxideand subsequent functionalization with glucose oxidase. *J. Phys. Chem. C* **113**, 14071–14075 (2009).

36. Mkhoyan, K. A. *et al.* Atomic and electronic structure of graphene-oxide. *Nano Lett.* **9**, 1058–1063 (2009).

37. Yin, Z. *et al.* Electrochemical deposition of zno nanorods on transparent reduced graphene oxide electrodes for hybrid solar cells. *Small* **6**, 307–312 (2010).

38. Shao, Y., Wang, J., Engelhard, M., Wang, C. & Lin, Y. Facile and controllable electrochemical reduction of graphene oxide and its applications. *J. Mater. Chem.* **20**, 743–748 (2010)

39. Ji, L. *et al.* Graphene oxide as a sulfur immobilizer in high performance lithium/sulfur cells. *J. Am. Chem.* Soc. **133**, 18522-18525 (2011).

40. Luo, Z., Lu, Y., Somers, L. A. & Johnson, A. T. C. High yield preparation of macroscopic graphene oxide membranes. *J. Am. Chem. Soc.* **131**, 898–899 (2009).

41. Chang, Y. *et al.* In vitro toxicity evaluation of graphene oxide on A549 cells. *Toxicol. Lett.* **200**, 201-210 (2011).

42. Eda, G., Mattevi, C., Yamaguchi, H., Kim, H. & Chhowalla, M. Insulator to semimetal transition in graphene oxide. *J. Phys. Chem. C* **113**, 15768–15771 (2009).

43. Yan, X., Chen, J., Yang, J., Xue, Q. & Miele, P. Fabrication of free-standing, electrochemically active, and biocompatible graphene oxide-polyaniline and graphene-polyaniline hybrid papers. *ACS Appl. Mater. Interfaces* **2**, 2521-2529 (2010).

44. Su, C. *et al.* Electrical and spectroscopic characterizations of ultra-large reduced graphene oxide monolayers. *Chem. Mater*. **21**, 5674–5680 (2009).

45. Xu, Y., Bai, H., Lu, G., Li, C. & Shi, G. Flexible graphene films via the filtration of water-soluble noncovalent functionalized graphene sheets. *J. Am. Chem. Soc.* **130**, 5856–5857 (2008).

46. Chen, Y., Zhang, X., Zhang, D., Yu, P. & Ma, Y. High performance supercapacitors based on reduced graphene oxide in aqueous and ionic liquid electrolytes. *Carbon.* **49**, 573-580 (2011).

47. Balapanuru, J. *et al.* A graphene oxide–organic dye ionic complex with DNA-sensing and optical-limiting properties. *Angew. Chem. Int. Ed*. **49**, 6549–6553 (2010)

48. Rourke, J. P. *et al.* The real graphene oxide revealed: Stripping the oxidative debris from the graphene-like sheets. *Angew. Chem. Int. Ed.* **50**, 3173-3177 (2011).

49. Sharma, S. *et al.* Rapid microwave synthesis of CO tolerant reduced graphene oxide-supported platinum electrocatalysts for oxidation of methanol. *J. Phys. Chem. C* **114**, 19459–19466 (2010).

50. Liu, S. *et al.* Antibacterial activity of graphite,graphite oxide,graphene oxide,and reduced graphene oxide:Membrane and oxidative stress. *ACS Nano* **5**, 6971-6980 (2011).

51. Zhu, J. *et al.* Facile synthesis of metal oxide/reduced graphene oxide hybrids with high lithium storage capacity and stable cyclability. *Nanoscale* **3**, 1084–1089 (2011).

52. Hu, W. *et al.* Protein corona-mediated mitigationof cytotoxicity of graphene oxide. *ACS Nano* **5**, 3693–3700 (2011).

53. Shi, W. *et al.* Achieving high specific charge capacitances in fe3O4/reduced graphene oxide nanocomposites. *J. Mater. Chem.* **21**, 3422–3427 (2011).

54. Bao, H. *et al.* Chitosan-functionalized graphene oxide as a nanocarrier for drug and gene delivery. *Small* **7**, 1569–1578 (2011).

55. Zhang, W. *et al.* Synergistic effect of chemo-photothermal therapy using pegylated graphene oxide. *Biomaterials* **32**, 8555-8561 (2011).

56. Liu, J. *et al.* Bulk heterojunction polymer memory devices with reduced graphene oxide as electrodes. *ACS Nano* **4**, 3987-3992 (2010).

57. Yang, S. *et al.* Folding/aggregation of graphene oxide and its application in Cu^{2+} removal. *J. Colloid. Interface. Sci.* **351**, 122-127 (2010).
